# Supplementary material for: NET-prism enables RNA polymerase-dedicated transcriptional interrogation at nucleotide resolution
Source: RNA Biol. 2019 Jun 3;16(9):1156–65. doi: 10.1080/15476286.2019.1621625 (PMC6693550; doi:10.1080/15476286.2019.1621625)
Supplement: Supplemental Material [file krnb-16-09-1621625-s001.zip › Supplementary information/SUPPLEMENTARY FIGURES.docx]

**SUPPLEMENTARY FIGURES**


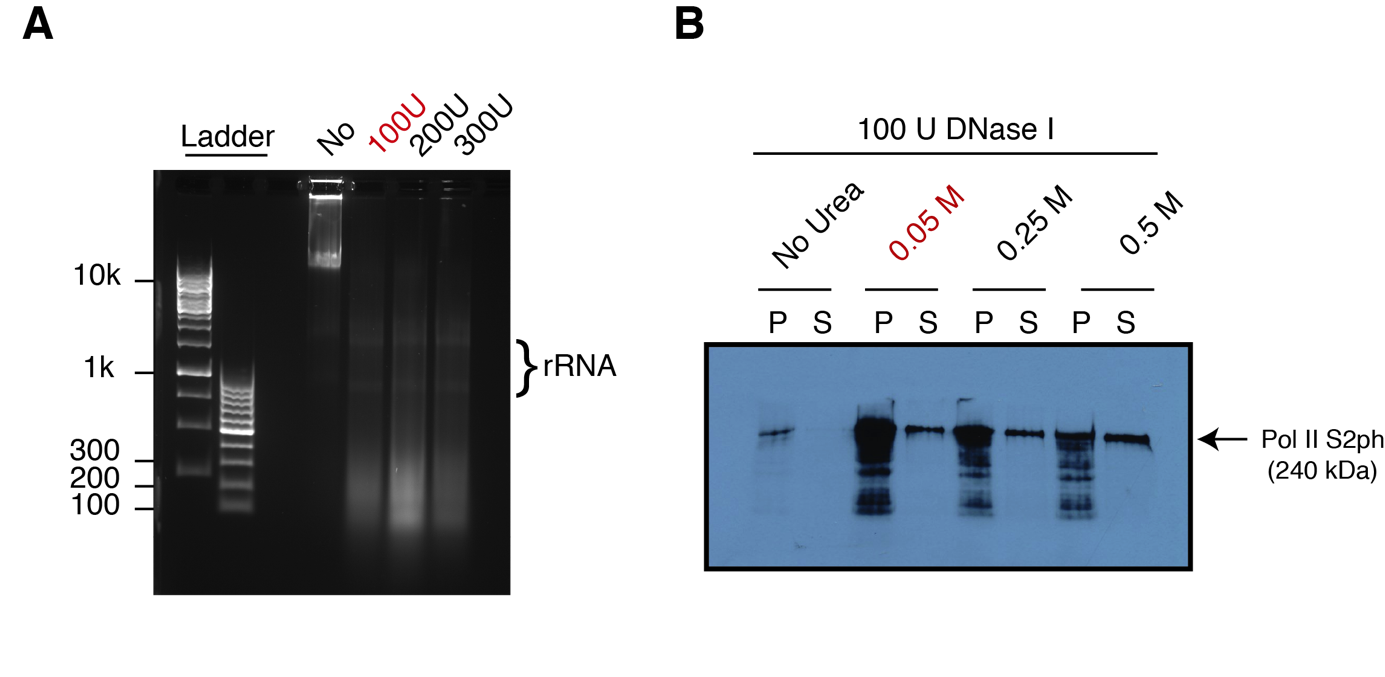


**Supplementary Figure 1:** Release of Pol II from chromatin via DNase I treatment. (A) Agarose gel (1%) showing the effect of different concentrations of DNAse I on DNA fragmentation. No difference is observed among treated conditions. (B) Western blot assessing Pol II release after treatment of 2x10^7^ mouse ES cells with different Urea concentrations (0, 0.05 M, 0.25 M, 0.5 M) in the presence of 100U of DNase I. The conditions highlighted in red (0.05 M Urea & 100U DNAse I) were used for the generation of all NET-prism libraries (P; Pellet, S; Supernatant).

**
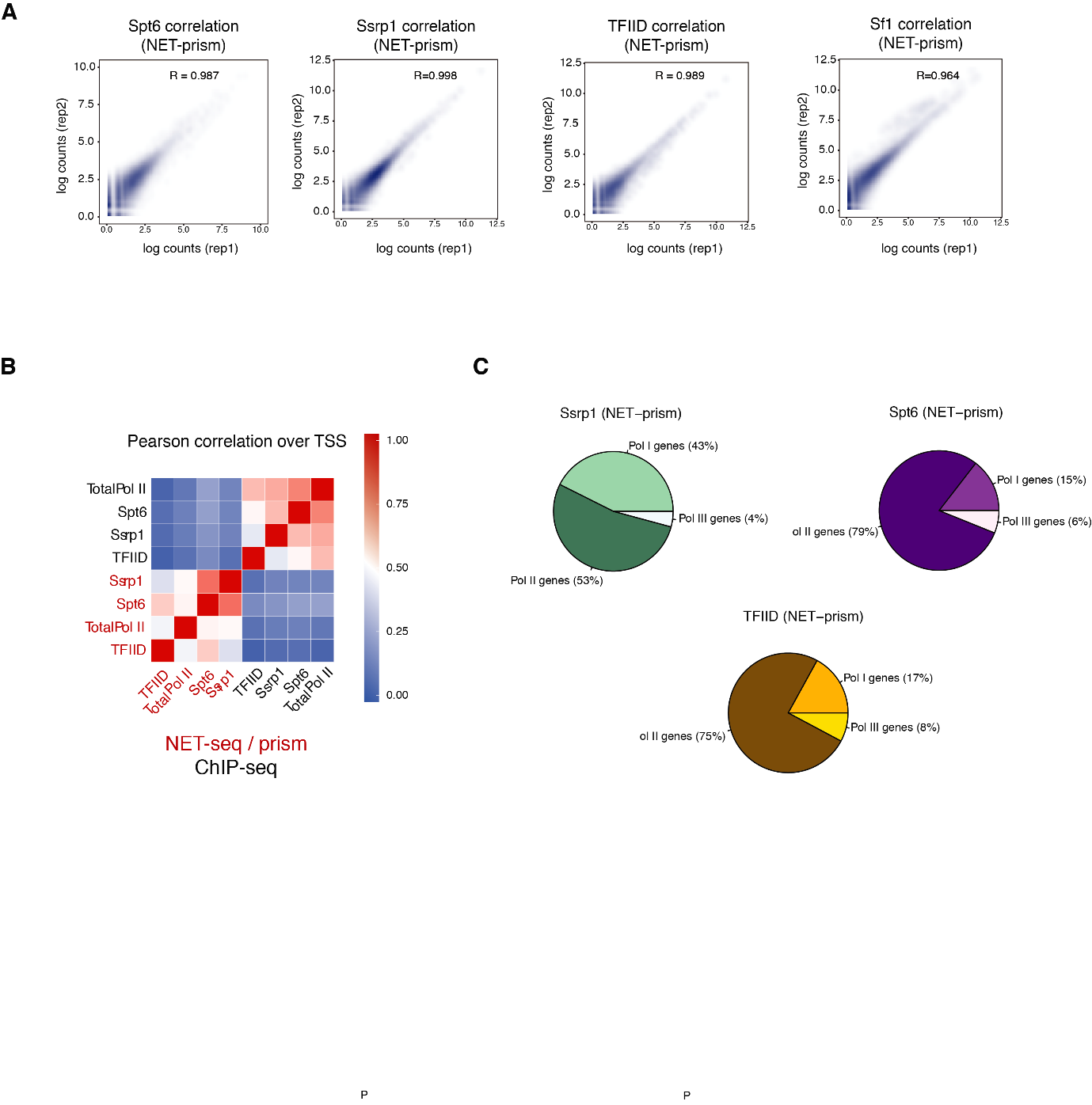
**

**Supplementary Figure 2:** Transcriptional activity assessed by NET-prism. (A) Correlation plots assessing reproducibility between replicates of Spt6, Ssrp1, TFIID, and Sf1 libraries (NET-prism). R corresponds to Pearson correlation. (B) Correlation heatmap of all NET-seq/prism (red) and ChIP-seq (black) libraries over promoter regions (± 500 bp around TSS) of all uniquely annotated genes (n = 12,737). (C) Distribution of reads mapping to Pol I, Pol II, Pol III transcribed RNAs for Ssrp1, Spt6, and TFIID NET-prism libraries in mESCs.


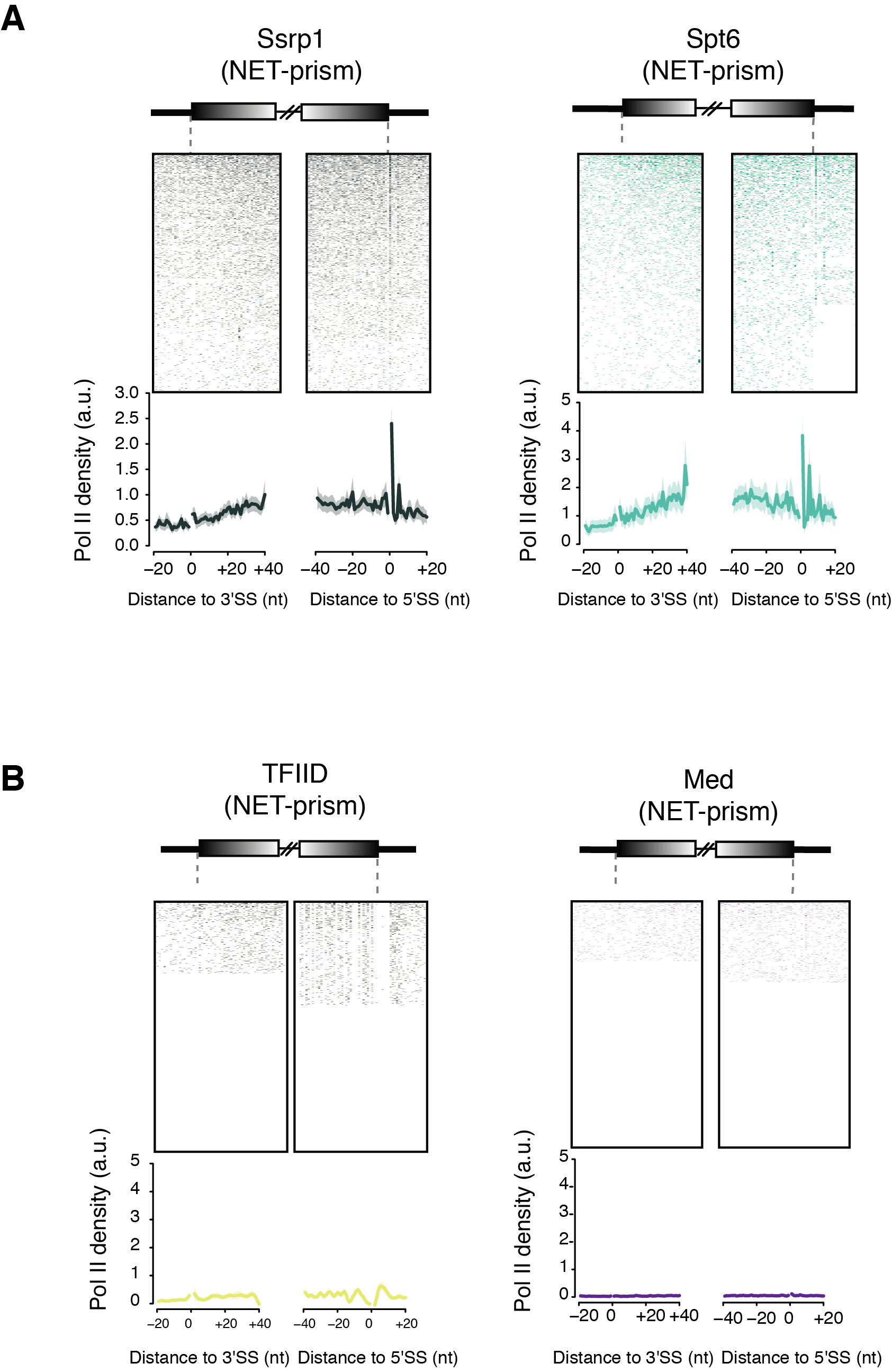


**Supplementary Figure 3:** No association of the PIC complex with co-transcriptional splicing. (A) Heatmaps and metaplots assessing polymerase pausing for Ssrp1, and Spt6 over exon boundaries (n = 2,586). Solid lines indicate the mean values, whereas the shading represents the 95% confidence interval. (B) Heatmaps and metaplots assessing polymerase pausing for TFIID, and Mediator over exon boundaries (n = 2,586). Solid lines indicate the mean values, whereas the shading represents the 95% confidence interval.


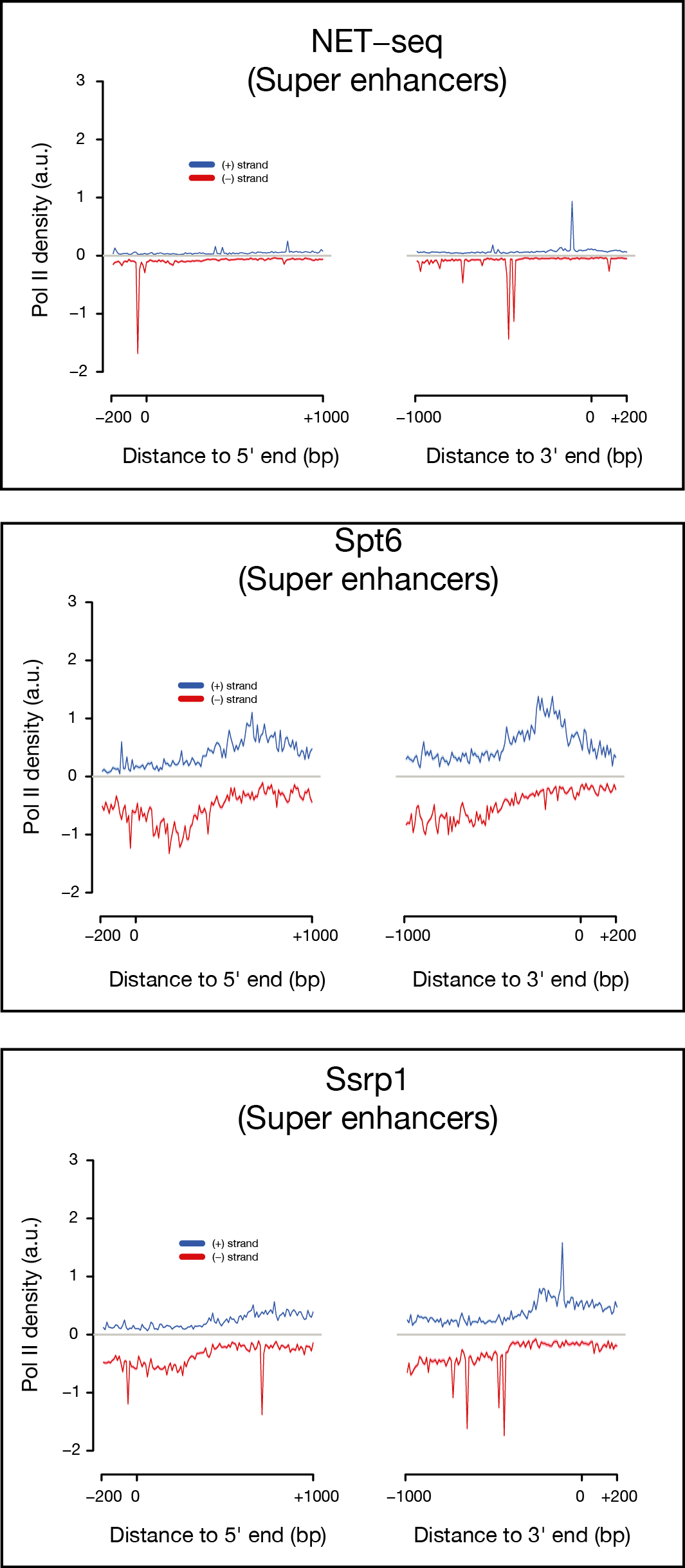


**Supplementary Figure 4:** Unique patterns of transcriptional pausing over super-enhancers. Metaplots assessing polymerase pausing for total Pol II, Pol II S5ph, Ssrp1, and, Spt6 over super-enhancers (n = 226). Solid lines indicate the mean values, whereas the shading represents the 95% confidence interval.

**NET-prism PROTOCOL**

**Quantitative purification of RNA polymerase by cell fractionation ● TIMING 45 min**

Cell fractionation is performed on ice or at 4 °C, with buffers freshly prepared on the same day. All buffers are precooled on ice before use.

Use RNase-free reagents and equipment.

**Preparation of antibody-conjugated beads** ● TIMING 2 h (or overnight)

1| Wash 50 µl of Dynabeads protein G with 1 ml of ice-cold IP buffer by vortexing at room temperature: repeat once more.

3| Resuspend the Dynabeads in 50 µl of ice-cold IP buffer.

4| Add ~10 µg of the appropriate antibody to washed beads. IgG can be used as control (optional).

5| Incubate the mixture on a tube rotator (12 r.p.m.) in a cold room for at least 2 h.

7| Briefly spin down tubes and place on ice until use.

**1|** Grow cells in a 15 × 2.5 cm dish in DMEM containing 10% (vol/vol) FBS, 100 U/ml penicillin and 100 µg/ml streptomycin until they are 90% confluent

**2|** Wash the cells twice with 10 ml of PBS buffer.

**CRITICAL STEP** Unless otherwise stated, perform all washing steps on ice.

**3|** Scrape or trypsinise cells into 1 ml of PBS buffer.

**4|** Determine the cell number by counting using a cell counter. Use 2×10^7^ cells as input for each cell fractionation. You will need in total 80-100×10^7^ cells for each IP.

**5|** Wash each sample of 2× 10^7^ cells with 10 ml of PBS.

**6|** Collect cells by centrifugation at 500g for 2 min at 4 °C.

**7|** Remove the supernatant by aspiration.

**CRITICAL STEP** It is important to remove the supernatant completely at this step. If the supernatant is not completely removed, the cytoplasmic lysis buffer (Step 8) will be diluted, which affects the cell lysis efficiency.

**8|** Add 200 µl of cytoplasmic lysis buffer and transfer it to an RNase-free 1.5-ml microcentrifuge tube. Cut a 1,000-µl pipette tip, and use it to pipette the sample up and down ten times.

**9|** Incubate the cell lysate on ice for 5 min.

**10|** Using a cut 1,000-µl pipette tip, layer the cell lysate onto 500 µl of sucrose buffer.

**11|** Collect cell nuclei by centrifugation at 16,000g for 10 min at 4 °C.

**12|** Remove the supernatant.

**13|** Add 100U of DNAse I in 100 µl of DNAse buffer (NEB) and place on ice for 20 min. Stop reaction by adding EDTA to a final concentration of 5 mM.

**14|** Wash nuclei with 800 µl of nuclei wash buffer.

**15|** Collect washed nuclei by centrifugation at 7,000g for 1 min at 4 °C.

**16|** Add 200 µl of nuclei lysis buffer, mix by pulsed vortexing and incubate the mixture on ice for 5 min.

**17|** Centrifuge the mixture at 18,500g for 2 min at 4 °C.

**18|** Combine supernatants from different fractionations (Final volume: 800 µl or 1000 µl)

**19|** Transfer to a 15ml falcon tube and add IP buffer (7.2ml or 9ml) to a final 1/10 dilution. Add RNase/protein inhibitors.

**20|** Add antibody–conjugated beads and mix immediately.

**21|** Incubate the samples on a tube rotator (12 r.p.m.) for 2 h in the cold room.

**22|** Spin the samples at 300g for 5 min at 4 °C.

**23|** Aspirate supernatant. Leave only 1ml behind and transfer to a new 1.5ml tube.

**24|** Place the tube on a magnetic rack and leave it for 1 min. Remove the remaining supernatant.

**27|** Wash the beads with 1 ml of ice-cold IP buffer by inverting tube, and repeat Step 24 three times (total 4 times) in the cold room, taking care to remove the supernatant after the final wash.

**28**| Add 700 µl of TRIzol reagent directly to the beads and mix well by pipetting.

**29**| Add 150 µl of chloroform. Mix well and leave for 2 min at room temperature.

**30**| Centrifuge at 18,000g for 30 min at 4 °C.

**31**| Carefully transfer the upper transparent layer (~300 µl) to a new tube without disrupting the phenol layer.

**32**| Add 900 µl 100% ethanol, 30 µl NaOAc (3M), and 2 µl GycoBlue.

**33**| Incubate at -80 °C for 1h.

**34**| Centrifuge at 18,000g for 30 min at 4 °C.

**35**| Remove supernatant and wash pellet once with 1ml 100% ethanol.

**36**| Centrifuge at 18,000g for 2 min at 4 °C.

**37**| Remove supernatant and wash pellet once with 1ml 70% ethanol.

**38**| Centrifuge at 18,000g for 2 min at 4 °C.

**39**| Completely remove supernatant and let pellet air dry on ice for 5 min.

**40**| Resuspend pellet in 13 µl of RNAse-free water.

**41**| Run 1 µl on a Tapestation to assess RNA size and concentration. Typical concentration is between 500 – 1000 ng/ µl. IPs for elongations factors (Spt6) yield large RNA fragments (>2000 nt) whereas PIC components yield lower (< 200 nt).

**42**| Parse samples into the same NET-seq library as previously described (Mayer & Churchman, 2016: *Nature Protocols*). Make the appropriate modifications as described below to allow a more efficient adaptor ligation and material recovery after ligation and cDNA synthesis.

- The random barcode was ligated overnight at 16 ºC to maximise ligation efficiency.
- Alkaline fragmentation of the ligated nascent RNA varies depending on the size of the RNA fragments obtained from each IP. IPs for Pol II S5ph, Pol II S2ph, Ssrp1, Spt6, and Sf1 yielded large RNA fragments and therefore the ligated nascent RNA was fragmented until all RNA transcripts were within the range of ~35-200 nucleotides. IPs for TFIID, and Mediator yielded fragments < 200 nt and therefore no fragmentation was performed.
- Maximum recovery of ligated RNA and cDNA was achieved from 15 % TBE-Urea (Invitrogen) and 10% TBE-Urea (Invitrogen), respectively, by adding RNA recovery buffer (Zymo Research, R1070-1-10) to the excised gel slices and further incubating at 70°C (1500 rpm) for 15 min. Gel slurry was transferred through a Zymo-Spin IV Column (Zymo Research, C1007-50) and further precipitated for subsequent library preparation steps.

**Buffers (x1 reaction)**

**µl**

**µl**

**Sucrose buffer**

**Cytoplasmic lysis buffer**

| 10% (vol/vol) NP-40 | **3.8** |
| --- | --- |
| 1 M Tris-HCl (pH 7.0) | **2.5** |
| 5 M NaCl | **7.5** |
| protease inhibitor mix (100×) | **2.5** |
| 1 mM α-amanitin | **6.2** |
| RNasin | **0.3** |
| RNase-free H2O | **227.2** |
| **Total** | **250** |

| 50% (wt/vol) filter-sterilized sucrose | **250** |
| --- | --- |
| 1 M Tris-HCl (pH 7.0) | **5** |
| 5 M NaCl | **15** |
| protease inhibitor mix (100×) | **5** |
| 1 mM α-amanitin | **12.5** |
| RNasin | **0.6** |
| RNase-free H2O | **211.9** |
| **Total** | **500** |

**µl**

**µl**

**Nuclei lysis buffer**

**DNase buffer**

| DNase buffer (x10) (NEB) | **10** |
| --- | --- |
| protease inhibitor mix (100×) | **1** |
| 1 mM α-amanitin | **2.5** |
| RNasin | **0.125** |
| RNase-free H2O | **86.375** |
| **Total** | **100** |

| 10% (vol/vol) NP-40 | **25** |
| --- | --- |
| 1 M HEPES (pH 7.5) | **5** |
| 0.1 M EDTA | **0.5** |
| 5 M NaCl | **5** |
| 10 M filtersterilized urea | **1.25** |
| 0.1 M filter-sterilized DTT | **1.25** |
| protease inhibitor mix (100×) | **2.5** |
| 1 mM α-amanitin | **6.2** |
| RNasin | **0.3** |
| RNase-free H2O | **203** |
| **Total** | **250** |

**IP buffer**

**ml**

| 1 M Tris-HCl (pH 7.0) | **5** |
| --- | --- |
| 5 M NaCl | **1** |
| 10% (vol/vol) NP-40 | **0.5** |
| RNase-free H2O | **93.5** |
| **Total** | **100** |
